# The Role of the DNA Methyltransferase Family and the Therapeutic Potential of DNMT Inhibitors in Tumor Treatment

**DOI:** 10.3390/curroncol32020088

**Published:** 2025-02-05

**Authors:** Dae Joong Kim

**Affiliations:** Department of Microbiology, Immunology & Cancer Biology, The University of Virginia, Charlottesville, VA 20908, USA; dk2ka@virginia.edu

**Keywords:** DNA methyltransferase (DNMT), DNA methylation, CpG island, 5-methylcytosine (5mC), tumor suppressor genes (TSGs), DNMT1i, guadecitabine

## Abstract

Members of the DNA methyltransferase (DNMT) family have been recognized as major epigenetic regulators of altered gene expression during tumor development. They establish and maintain DNA methylation of the CpG island of promoter and non-CpG region of the genome. The abnormal methylation status of tumor suppressor genes (TSGs) has been associated with tumorigenesis, leading to genomic instability, improper gene silence, and immune evasion. DNMT1 helps preserve methylation patterns during DNA replication, whereas the DNMT3 family is responsible for de novo methylation, creating new methylation patterns. Altered DNA methylation significantly supports tumor growth by changing gene expression patterns. FDA-approved DNMT inhibitors reverse hypermethylation-induced gene repression and improve therapeutic outcomes for cancer. Recent studies indicate that combining DNMT inhibitors with chemotherapies and immunotherapies can have synergistic effects, especially in aggressive metastatic tumors. Improving the treatment schedules, increasing isoform specificity, reducing toxicity, and utilizing genome-wide analyses of CRISPR-based editing to create personalized epigenetic therapies tailored to individual patient needs are promising strategies for enhancing therapeutic outcomes. This review discusses the interaction between DNMT regulators and DNMT1, its binding partners, the connection between DNA methylation and tumors, how these processes contribute to tumor development, and DNMT inhibitors’ advancements and pharmacological properties.

## 1. Introduction

**Figure 1:**
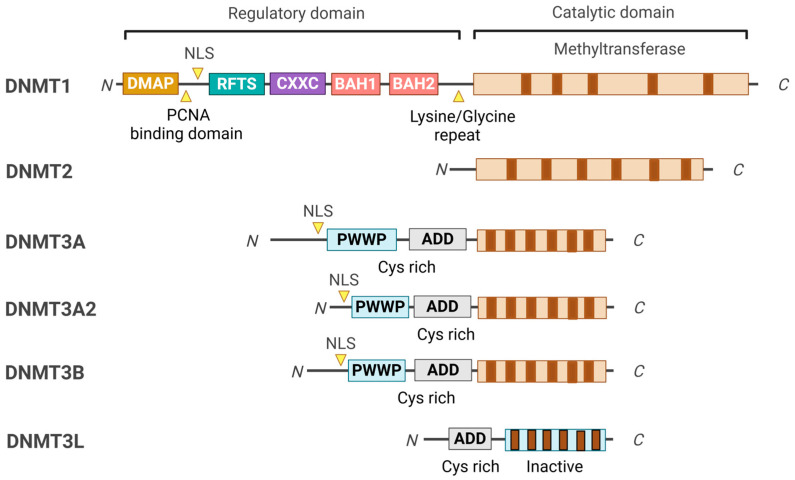
Domain structure of DNMT family members. The conserved domains of the DNMT family members are represented in different colors. All DNMTs share a common catalytic domain; however, DNMT2 lacks the regulatory domain, which sets it apart from the other DNMTs. DNMT3L is a catalytically inactive variant of DNMT3 that is missing the N-terminal portion of the regulatory domain, including the PWWP and the C-terminal part of the catalytic domain. Instead, DNMT3L functions as a co-factor for DNMT3A and DNMT3B.

The key feature of epigenetic regulation in living organisms is DNA methylation, which is a critical factor in physiological and disease processes [1]. Identifying the fifth base of mammalian DNA, 5-methylcytosine (5mC), located in gene promoters, is crucial for regulating gene expression without altering the underlying nucleotide sequence and has significantly transformed various fields of biomedical research [2]. The primary regulators of DNA methylation are DNA methyltransferases (DNMTs) comprising DNMT1, DNMT2, DNMT3A, DNMT3B, and DNMT3L (Figure 1) [3]. Among these, DNMT1, DNMT3A, and DNMT3B are the canonical cytosine-5 DNA methyltransferases responsible for adding methylation marks to genomic DNA. Conversely, DNMT2 and DNMT3L are considered non-canonical members of the DNMT family because they lack catalytic DNMT activity. Nonetheless, DNMT2 and DNMT3L display significant sequence conservation with the canonical DNMT enzymes, representing evolutionary adaptations of the original DNMT genes [4]. DNMTs are central to this process, which utilizes S-adenosylmethionine (SAM) as a methyl group donor and employs a base-flipping mechanism, allowing them to rotate the target base into their catalytic pocket [5]. This epigenetic modification profoundly impacts gene expression by influencing the accessibility of DNA to transcription factors and other regulatory proteins. In eukaryotic genomes, the predominant form of methylation is 5mC, primarily located in CpG dinucleotides [6,7]. The symmetrical distribution of CpG methylation marks on both DNA strands facilitates the post-replicative maintenance of these methylation patterns, serving as a critical component of epigenetic regulation [8,9].

In vertebrates, the first knockout of the DNMT family was *Dnmt1* [10], which resulted in embryonic lethality (Table 1). The deletion of *Dnmt3a* impairs postnatal development, while *Dnmt3b* deletion also leads to embryonic lethality [11]. Mice with *Dnmt2* mutation are viable but exhibit abnormal hematopoiesis [12,13]. Deficiencies in DNMT1 and DNMT3 result in significant developmental defects in mice. In contrast, some organisms, such as *Caenorhabditis elegans* and *Saccharomyces cerevisiae*, lack DNMT genes [14]. Triple-knockout mouse embryonic stem (ES) cells lacking DNMT1, DNMT3A, and DNMT3B continue to proliferate normally [15], but DNMT1-deficient ES cells fail to differentiate [16]. Interestingly, undifferentiated human ES cells lacking DNMT1 are not viable, likely due to differences in pluripotent states [3].

Recently, there has been growing interest in the sporadic occurrence of non-CpG methylation, such as at CpA and CpT sites, especially in specific cell types like embryonic stem cells and neurons, which display increased de novo methylation activity [20]. The current advanced understanding of DNA methylation shows that the patterns of DNA methylation are not restricted to CpG sites during de novo methylation. Initially, this nonspecific de novo methylation was thought to merely result from the activity of DNMT3, which does not exhibit a preference for CpG dinucleotides [21,22,23]. Non-CpG methylation may serve an independent epigenetic role, particularly evident in neuronal maturation [24]. For example, methylation occurring at sites beyond the traditional CpG dinucleotide can attract methyl-CpG-binding protein 2 (MECP2), which acts as a critical transcriptional repressor, especially for longer genes that are involved in neuronal functions [25].

**Figure 2:**
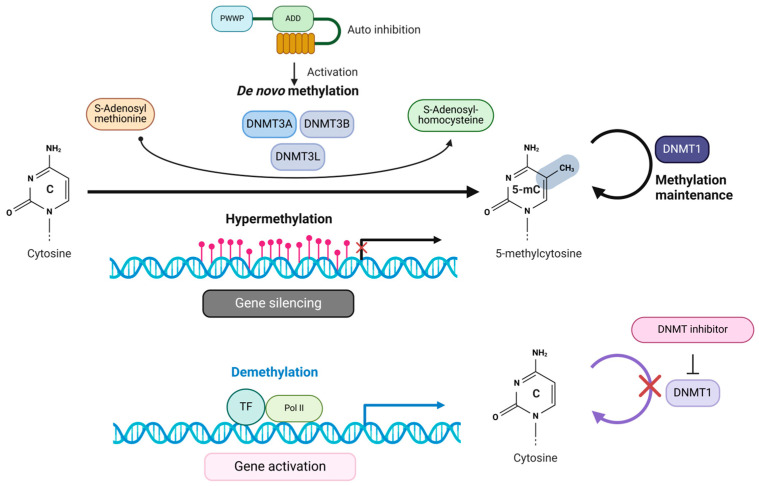
Methylation of cytosine and its effect on transcriptional regulation by DNMTs. DNMTs facilitate the methylation of cytosines within CpG dinucleotides located in gene promoter regions. They achieve this by transferring methyl groups from the methyl donor, SAM, forming 5mC and SAH. The hypermethylation of cytosines leads to the suppression of gene transcription. Conversely, DNMT inhibitors work by demethylating CpG island promoters, promoting the binding of transcription factors (TFs). As a result, the gene becomes activated, allowing transcription to occur.

The interplay between DNA methylation and demethylation processes adds adaptability to the typically stable DNA code, allowing controlled gene expression adjustments in response to external and internal cues. These flexible yet generally stable processes are increasingly valuable for distinguishing between healthy and diseased conditions. In the context of cancer, despite genome-wide hypomethylation, hypermethylation of CpG islands (changes in the overall levels of 5mC and 5hmC) is not only useful for early detection but also provides insights into the underlying mechanisms of cancer development and patient prognosis. These shifts in DNA methylation are fundamental to tumorigenesis and the preservation of tumor suppressor genes (TSGs) [6,7]. Several key characteristics define the abnormal DNA methylation observed in tumor cells, including the overexpression of DNMT, which results in increased catalytic activity within these cells. Additionally, a decline in DNA methylation levels occurs near highly repetitive sequences, contributing to chromosomal instability and the reactivation of transposon elements (TEs) [26]. Furthermore, hypermethylation in the promoters of TSGs results in their suppressed expression, further exacerbating tumor development and progression [17]. DNMT1 maintains aberrant DNA methylation patterns during DNA replication, perpetuating epigenetic dysregulation and contributing to tumorigenesis. DNMT1 cooperates with several binding partners to sustain DNA methylation, including UHRF1, Sp1, PCNA, and histone methyltransferases. This collaboration directly links DNMT1 to oncogenic processes and the silencing of TSGs [27]. In contrast, DNMT3A and DNMT3B are primarily responsible for de novo methylation, focused on establishing new methylation marks rather than preserving existing ones (Figure 2) [28,29]. Given DNMT1’s pivotal role in maintaining aberrant methylation in cancer cells, it has emerged as a critical target for therapeutic interventions against epigenetic stability of tumorigenesis, as altered DNA methylation can contribute to cancer progression (See also, Box 1).

DNMT1 undergoes post-translational modifications (PTMs) that significantly impact its function. For instance, LSD1 can demethylate Lysine 1094, while SET7 can methylate lysine 142 [30,31]. Additionally, AKT1 phosphorylates DNMT1 at serine 143, which inhibits methylation at lysine 142 and prevents its degradation [27], thereby linking DNMT1 stability to the PI3K–AKT–mTOR pathway. DNMT2 may also be phosphorylated at serine 215 and 219 by ATM, facilitating the relocation of stress granules [13]. Moreover, DNMT3A can be phosphorylated by casein kinase 2 at serine 386 and 389, enhancing its targeting to heterochromatin [32]. These alterations in DNA methylation are integral to tumorigenesis and the maintenance of TSGs.

Box 1 **Biological effect of DNA methylation.**

The change in DNA methylation patterns plays a significant role in cancer development. The altered DNA methylation patterns affect various genes in our body and influence essential biological processes such as genome stability, inflammation, cell cycle regulation, and metabolism [33]. The changes typically involve two main processes: hypomethylation (a decrease in methylation across the genome) and hypermethylation (an increase in methylation in specific regions, such as CpG islands) causing cancer.

**1. Hypomethylation**

(1) Genome Stability: a. LINE-1 and Alu Sequences: The loss of methylation in these regions heightens genomic instability by activating transposable elements and causing insertional mutagenesis [34]. b. Centromeric and Telomeric Regions: Hypomethylation in these areas contributes to chromosomal instability and is associated with shorter telomeres related to aging and cancer [35].

(2) Oncogenes: Activating RAS family genes through hypomethylation can contribute to cancer development, a common age-related disease [36].

(3) Amyloid Precursor Protein (APP) hypomethylation may lead to increased APP expression, resulting in excessive amyloid-beta (Aβ) production, which forms the plaque characteristic of Alzheimer’s disease [37].

(4) Hypomethylation of SNCA (α-synuclein) is frequently observed in the promoter region of the SNCA gene in Parkinson’s disease (PD). This hypomethylation increases α-synuclein expression, contributing to its aggregation and the formation of Lewy bodies, a hallmark of PD [38].

(5) Stem Cell Functionality: Hypomethylation is crucial for keeping pluripotency-associated genes, such as OCT4, SOX2, and NANOG, active. During the differentiation process, there is a transition towards hypomethylation in lineage-specific genes, allowing their activation [39]. Understanding the specific roles of hypermethylation and hypomethylation is essential for enhancing stem cell-based therapies in regenerative medicine.

**2. Hypermethylation**

(1) Tumor Suppressor Genes: (a) SOCS1 (Suppressor of Cytokine Signaling 1) regulates cytokine signaling and inhibits tumorigenic pathways, specifically the JAK-STAT signaling pathway, and is frequently hypermethylated in cancers [40]. (b) CDKN2A (p16, p14ARF) controls cell cycle progression; its silencing leads to unchecked cell division and is associated with aging and cancer [41]. (c) PTEN is a phosphatase that plays a pivotal role in negatively regulating the PI3K/AKT pathway, preventing uncontrolled cell growth [42]. (d) MLH1 is a crucial mismatch repair (MMR) gene, and its silencing results in microsatellite instability (MSI) observed in cancers [43]. (e) TP53 regulates DNA repair processes, apoptosis, and cancer. TP53 is rarely hypermethylated in cancers [44]. The nuclear entry of p53 and autophagy in cancer cells are associated with a circular RNA circ-Dnmt1, particularly in the epigenetic regulation of autophagy-related genes [45].

(2) HLA genes can impair antigen presentation and diminish immune responses. Additionally, hyper-methylation of anti-inflammatory or immune-regulatory genes can lead to dysregulated production of pro-inflammatory cytokines, contributing to inflammation [46].

(3) DNA Repair Genes: Genes involved in DNA repair often exhibit hypermethylation in aging cells. The silencing of BRCA1, MLH1, and MGMT diminishes DNA repair efficiency, resulting in genomic instability and contributing to the aging process and cancer [47].

(4) Regulation of cell cycle genes in cancer development: (a) Hypermethylation: RB1 regulates the cell cycle checkpoint [48]. (b) Hypomethylation: E2F targets transition from the G1 to the S phase [49].

(5) SIRT1: Hypermethylation of the SIRT1 promoter region can suppress its expression, diminishing its role in stress response and energy metabolism, which is related to metabolic disorders, chronic inflammation, aging, and cancer development [50].

(6) Hypermethylation of PPARGC1A has been associated with decreased expression, which adversely affects mitochondrial biogenesis and energy homeostasis, commonly observed in age-related metabolic disorders and cancer [51,52].

**3. Dysregulated methylation**

(1) IL-6 and TNF-alpha: Both hypo- and hypermethylation can influence the expression of these pro-inflammatory cytokines. Hypomethylation of promoter regions can lead to increased expression, while hypermethylation of regulatory regions can result in reduced expression. However, chronic low-grade inflammation associated with aging and cancer is often linked to diminished regulation of these genes. This suggests a dysregulation of methylation patterns, including hypermethylation of regulatory regions and hypomethylation of promoters [53,54].

(2) HOX genes: (a) Hypermethylation: During aging or in certain diseases, hypermethylation of HOX gene promoters can lead to the silencing of these genes. This silencing disrupts normal cellular differentiation and tissue maintenance, contributing to dysfunction associated with aging or diseases like cancer. For example, hypermethylation of specific HOX genes has been observed in tumors, resulting in abnormal differentiation [55]. (b) Hypomethylation: Hypomethylation in HOX genes’ regulatory regions can cause inappropriate reactivation or misexpression [56]. This may result in the undesired activation of developmental pathways in adult tissues, leading to dysregulation that could contribute to cancer progression.

Many conventional chemotherapies and targeted inhibitors work by directly killing rapidly dividing cells, which leads to cytotoxicity in target tissues. This often involves pathways related to DNA replication and damage. In contrast, inhibitors of DNA methyltransferase (DNMTi) do not induce systemic cytotoxicity; instead, they reprogram the epigenetic landscape by addressing abnormal DNA methylation patterns in cancers. DNMT1 inhibitors can demethylate hypermethylated regions of tumor-suppressor genes and other regulatory genes, whose silencing contributes to malignant transformation. DNMTi plays a significant role in tumor immunotherapy. On the one hand, DNMTi can enhance tumor antigen presentation by upregulating the expression of tumor-associated antigens (TAAs) and increasing major histocompatibility complex class I (MHC I) expression through the inhibition of DNA hypermethylation in the promoter of MHC I [57]. Additionally, DNMTi can remodel the chromatin of tumor cells by inhibiting DNA methylation and histone modification [58]. On the other hand, DNMTi can improve the function of cytotoxic T lymphocytes (CTLs) by upregulating the expression of cytokines such as IL-2, IFN-γ, CCL5, CXCL9, and CXCL10 [2,59]. Moreover, DNMTi can promote immune responses by enhancing NK cell-mediated cytotoxic effects and modulating immunosuppressive cells. Overall, DNMTi can enhance the effectiveness of immunotherapies and work synergistically with other targeted agents in combinatory therapy. Although significant progress is being made in this research area, the precise mechanisms underlying these changes remain incompletely understood, indicating the more specific drug mechanism and the potential involvement of additional factors in determining specific methylation profiles in cancer. This review discusses the relationship between DNA methylation and tumors and the ongoing research advancements, underlying mechanisms, and pharmacological properties of DNMT inhibitors to promote their further development as effective anti-tumor agents.

## 2. DNMT Domain Organizations and Their Functions

DNMTs are classified into three main classes: DNMT1, DNMT2, and DNMT3A/3B/3L, each serving distinct roles in DNA methylation processes [3]. Among these, DNMT1 is a major member and crucial in preserving DNA methylation patterns during DNA replication, ensuring the faithful inheritance of epigenetic marks. DNMT1 possesses two isoforms, DNMT1A and DNMT1B, which differ in their N-terminal regions due to alternative splicing (Figure 1). The N-terminal region of DNMT1 is essential for its regulatory functions, localization, and interactions with other proteins, directing DNMT1 to specific chromatin sites during DNA replication while maintaining precise DNA methylation patterns [60]. This region includes several specialized domains: 1. The DMAP1 Binding Domain, which facilitates interaction with DNA methyltransferase-associated protein 1 (DMAP1) and histone deacetylase 2 (HDAC2) for chromatin remodeling and transcriptional repression [61]. 2. The PCNA Binding Domain is crucial for targeting DNMT1 to replication foci through its interaction with proliferating cell nuclear antigen (PCNA) during the S phase of the cell cycle, ensuring synchronization of methylation with DNA replication [62]. 3. The Nuclear Localization Signal (NLS) guides DNMT1 to the nucleus, where it performs its essential role in DNA methylation [63]. 4. The Replication Foci-Targeting Sequence (RFTS) Domain, necessary for localization to replication sites, also serves as an autoinhibitory domain to prevent unscheduled methylation [64]. 5. The CXXC Domain, a conserved zinc-finger domain that binds to unmethylated CpG sites, ensuring DNMT1 accurately targets hemimethylated sites, along with two bromo-adjacent homology (BAH) domains that contribute to DNMT1’s structural stability and facilitate connections with other chromatin-associated proteins [65,66]. The C-terminal catalytic domain of DNMT1 is important for transferring a methyl group from SAM to the cytosine residues of CpG dinucleotides. This region is highly conserved among DNMT enzymes, reflecting its critical role in enzymatic activity. Alterations in this domain can lead to impaired methylation capabilities and are associated with various diseases, including cancer and neurological disorders [4].

DNMT2 was initially characterized as a DNMT due to a cytosine-5 DNMT domain [23]. However, subsequent biochemical assays have demonstrated minimal DNA methylation activity [13]. The cellular localization of DNMT2 is primarily in the cytoplasm, which complicates its proposed function in DNA methylation [13]. Despite this, DNMT2 shows a notable phylogenetic relationship with the DNMT family [13]. DNMT2 is recognized as a tRNA methyltransferase that utilizes the catalytic mechanism of DNMTs to transfer a methyl group from SAM (AdoMet) to the C38 position of tRNA. This process results in m5C methylation and produces S-adenosylhomocysteine (AdoHcy; SAH) as a by-product. The C38 tRNA methylation, located near the anticodon loop of tRNAs, is associated with post-transcriptional gene regulation and protein translation, thereby expanding the regulatory roles of the DNMT family [67].

The DNMT3 family consists of three members: DNMT3A, DNMT3B, and DNMT3L. These proteins are primarily responsible for de novo DNA methylation in CpG and non-CpG sites, establishing the essential methylation patterns necessary for embryonic development and neurons that establish new methylation patterns essential for proper organismal development [29,68]. DNMT3A exists in two isoforms: DNMT3A1, which has an extended N-terminal region, and DNMT3A2, the shorter variant. DNMT3A is widely expressed and is critical in DNA methylation during early development and in adult tissues (Figure 1). Mutations in the DNMT3A gene can lead to developmental disorders, such as Tatton–Brown–Rahman syndrome, and are also associated with certain cancers, including acute myeloid leukemia [69,70]. The rarity of disease-associated mutations suggests strong selection against loss-of-function mutations. These mutations are often heterozygous missense variants resulting in hypomorphic alleles, making it challenging to replicate disease phenotypes in knockout mouse models [71]. DNMT3B works alongside DNMT3A, focusing on methylation in centromeric and pericentromeric regions. Mutations in DNMT3B are linked to immunodeficiency, centromeric instability, and facial anomalies (ICF) syndrome [72]. Both DNMT3A and DNMT3B share a similar structural organization, consisting of three essential domains: the Pro-Trp-Trp-Pro (PWWP) domain, the ATRX-DNMT3L-DNMT3A (ADD) domain, and the catalytic domain. The PWWP domain specifically recognizes methylated histones, such as H3K36me3, which helps localize these enzymes to specific chromatin regions [73]. The ADD domain binds to unmodified histone tails, while the catalytic domain performs methylation. DNMT3B mutations are associated with immunodeficiency, centromeric instability, and facial anomalies (ICF) syndrome [23,74]. Approximately half of patients with ICF syndrome have DNMT3B mutations, while the other half have mutations in genes indirectly affecting DNMT3B-mediated methylation, such as LSH [75]. Another member, DNMT3C, is found in mouse germ cells and shares the ADD and catalytic domains but also lacks a PWWP domain, indicating a specialization of function [11]. This enzyme shares significant sequence similarities with DNMT3B and is crucial in methylating younger retrotransposons [75]. DNMT3C also contributes by selectively methylating young transposons [76]. This highlights the intricate dynamics of DNMTs in transposon silencing. The other member, DNMT3L, is not catalytically active because it lacks a catalytic domain (Figure 1). Instead, it functions as a regulatory protein that enhances the activities of DNMT3A and DNMT3B [28]. Although DNMT3L does not possess a PWWP domain and cannot recognize chromatin marks, it is a cofactor that modulates the de novo methylation activities of DNMT3A and DNMT3B [77].

## 3. Recruitment of DNMTs to Gene Promoters

The targeting of DNMTs to specific loci for aberrant hypermethylation in cancer is not fully understood. One proposed mechanism involves DNA damage-dependent methylation of CpG islands. Genotoxic stress, such as oxidative stress, can induce DNA damage that leads to the accumulation of DNMTs and other complexes at CpG-rich promoters, resulting in transcriptional repression [78,79]. Additionally, the protein OGG1, which repairs oxidative damage, can recruit DNMT, EZH2, and G9a to damaged sites via the CHD4–NuRD complex, thus contributing to heterochromatin formation [80]. DNMTs facilitate the methylation of cytosines within CpG dinucleotides located in gene promoter regions. They achieve this by transferring methyl groups from the methyl donor, SAM, forming 5mC and SAH. The hypermethylation of cytosines leads to the suppression of gene transcription. Conversely, DNMT inhibitors work by demethylating CpG island promoters, promoting the binding of transcription factors (TFs). As a result, the gene activates, allowing transcription (Figure 2).

## 4. DNMT1 Binding Partners

In Section 4 and Section 5, we will discuss DNMT1’s binding partners and regulators. DNMT1 is a critical enzyme responsible for maintaining DNA methylation patterns and regulating gene expression, particularly in tumor suppressor genes (TSGs) such as p16INK4 and BRCA1, frequently silenced in cancer. This enzyme interacts with several key binding partners and regulators that play pivotal roles in its function. These interactions help DNMT1 bind to regions that require maintenance methylation and influence its stability by forming complexes with DNMT1 and other associated proteins, thereby linking DNA methylation processes to histone modifications.

Section 6 will discuss recent progress in DNMTi in therapy efficacy and clinical trials. DNMTi, including azacitidine and decitabine, modulates cytosine methylation, promoting the reactivation of tumor suppressor genes and enhancing immune responses against cancer. While decitabine has proven effective for treating myelodysplastic syndromes, the novel DNMT1 inhibitor guadecitabine shows promise in targeting solid tumors and addressing some of the limitations faced by earlier inhibitors.

DNMT1 regulates gene expression by binding to CpG islands in various gene promoters. It is crucial in maintaining DNA methylation patterns during replication (Figure 2) [27]. This binding can result in the transcriptional silencing of important TSGs such as p16INK4, p14ARF, and the adhesion protein CDH1 (E-cadherin), as well as GSTP1 (glutathione S-transferase pi1) and BRCA1 (which is involved in DNA repair). All of these genes are affected by DNMT1-mediated DNA methylation [81,82,83]. The stability of DNMT1 is regulated by TRAF6 (TNF receptor-associated factor 6), an E3 ubiquitin ligase. TRAF6-mediated ubiquitination of DNMT1 leads to its degradation, impacting DNA methylation patterns and gene expression [84]. DNMT1 forms a complex with the Retinoblastoma tumor suppressor protein (Rb), E2F1, and Histone Deacetylase 1 (HDAC1) to deacetylate histones and repress gene expression. This establishes a link between DNA methylation and histone acetylation [85]. This complex is believed to maintain DNA methylation patterns by suppressing genes susceptible to DNA demethylation.

Recent studies suggest a potential relationship between MLH1 and DNMT1 that may influence tumor antigenicity, triggering immune responses that can target and eliminate cancer cells. In colorectal cancer cells, decreased expression of MLH1 has been correlated with increased DNA methylation and reduced tumor antigenicity [43]. It is proposed that elevated levels of DNMT1 in cancer cells may lead to the downregulation of MLH1, resulting in an overall decrease in tumor antigenicity [86]. Furthermore, TIMP3, recognized as a tissue inhibitor of metalloproteinases and important for extracellular matrix remodeling, is often transcriptionally silenced in cancer cells due to DNA methylation driven by DNMT1. Additionally, DNMT1 can silence the CDKN2A TSG [81].

An intriguing relationship also exists between MAGE (Melanoma-Associated Antigen) proteins and DNMT1, which significantly influences gene expression and may play a critical role in tumor development. MAGE proteins are cancer-testis antigens highly expressed in melanoma and other cancers but are generally absent in normal tissues, except for the testes [87]. Also, DNMT1 has been shown to collaborate with the protein EZH2 to overcome melanoma resistance to immunotherapy [88]. 

G9a and DNMT1 by CM272 severely reduce methylation of SCARA5 and AOX1promoter and increase the efficacy of non-small cell lung cancer (NSCLC) [89]. The following section details key factors known to DNMT1 binding partners.

(1) MBDs

Methyl-CpG-binding domain proteins (MBDs) include key members like MeCP2 and MBD1–4, essential for interpreting methylated DNA and mediating DNA methylation by recruiting DNMT1 [90]. The MBD2/MBD3 heterodimer specificallybinds to hemimethylated DNA during the late S phase, enhancing DNMT1 recruitment to ensure accurate methylation propagation [90]. Interestingly, MeCP2 interacts with histone deacetylase 1 (HDAC1) and mSIN3A to regulate histone acetylation, impacting gene expression [91]. However, DNMT1 competes with mSIN3A for MeCP2’s transcription repression domain, leading to a functional shift. When DNMT1 binds, it disrupts the MeCP2/HDAC1/mSIN3A complex, promoting further methylation [92].

(2) CFP1

CFP1 and DNMT1 interact in living organisms through specific regions of the CFP1 protein, which include Zn2^+^ binding and chromatin targeting domains (PHD1, CXXC, PHD2) [93]. The CXXC domain binds unmethylated CpG dinucleotides, while the PHDs may associate with methylated histones. A mutation disrupting CFP1’s interaction with certain complexes does not affect its interaction with DNMT1, indicating distinct interaction pathways. Although CFP1 and DNMT1 fragments did not directly interact in vitro, posttranslational modifications or other proteins may mediate their interaction. Furthermore, cells lacking CFP1 show more severe defects than those lacking global cytosine methylation, suggesting additional factors contribute to these defects. The overlap between CFP1 and DNMT1 interaction regions suggests CFP1 may help target DNMT1 to chromatin, especially during transitions between euchromatin and heterochromatin [93]. This interaction could facilitate gene repression and contribute to the global euchromatin architecture observed in CFP1-deficient cells, potentially explaining their differentiation and survival defects.

(3) DMAP1 (DNMT-associated protein1)

DMAP1 (DNMT-associated protein 1) is a co-repressor in DNA methylation inheritance. It interacts with DNMT1 during the early and late phases of DNA replication, aiding in the recruitment of PCNA (proliferating cell nuclear antigen) [94]. The DNMT1/DMAP1 complex represses genes targeted by the glucocorticoid receptor, relying on DMAP1’s recruitment of DAXX, a protein involved in apoptosis and transcriptional repression. HDAC2 (histone deacetylase 2) can also join the DNMT1/DMAP1 complex to promote gene silencing. RGS6 (regulator of G protein signaling 6) competes with DNMT1 for DMAP1, disrupting their interaction. While these complexes do not have a specific DNA sequence preference, they may be linked to different DNA methylation profiles. Disruption of DNMT1 interactions can lead to global DNA hypomethylation and tumor growth. Notably, inhibiting the DNMT1/DMAP1 interaction has enhanced glioma cell response to temozolomide, suggesting the potential for targeting these complexes in chemotherapy [95].

(4) UHRF1

DNA replication ensures the accurate replication of these patterns by transferring methylation marks from the parent DNA strand to the daughter strand [10]. During DNA replication, DNMT1 collaborates with UHRF1 (Ubiquitin-like containing PHD and RING Finger domains 1), a key factor for sustaining methylation. UHRF1 contains a SET and RING-associated (SRA) domain, which recognizes hemimethylated DNA, marking it for recruitment by DNMT1 [96]. This collaboration ensures that newly synthesized DNA strands inherit the methylation patterns of their parent strands. The knockout phenotype of UHRF1 or DNMT1 showed a global reduction of DNA methylation and early death in the gastrulation stage [61,97].

(5) Sp1 (Specificity protein 1)

Sp1 can directly interact with DNMT1 in GC-rich promoter regions, leading to the methylation of CpG sites in those areas, which results in transcriptional repression. This interaction between Sp1 and DNMT1 can contribute to suppressing P16INK4a and RASSF1A, ultimately promoting tumorigenesis [98,99].

(6) PCNA (Proliferating Cell Nuclear Antigen)

DNMT1 interacts with the PCNA binding motif, called the PIP box at the N-terminal region (residue 164–171), the 310-helix (residues 167–171), and the C-terminal stretch (residues 172–174), during S phase [100,101]. The major DNMT1/PCNA/UHRF1 complex is predominantly active during the S phase of the cell cycle [102]. DNMT1 appears to interact primarily with Sp1 during the G1 and G2 phases, while interactions with DNMT1/P53 and DNMT1/E2F3 are mainly observed during the G2 and S/G2 phases, respectively, in U251 cells [103].

(7) G9a/GLP (Histone Methyltransferases)

G9a catalyzes the demethylation of H3K9 (H3K9me2) and induces transcriptional repression. G9a is known to interact with DNMT1 to coordinate DNA methylation and histone H3K9 methylation, creating a synergistic repressive chromatin structure. Their interaction plays an important role in cancer development [104].

(8) RUNX1-MTG8

In acute myeloid leukemia, the t(8;21)(q22.q22) translocation results in the formation of the chimerical protein RUNX1-MTG8, which acts like oncogenic TF. In normal cells, RUNX1 attaches to the enhancer motif TGT/CGGT. On the other hand, MTG8 functions as a transcriptional repressor, capable of interacting with various co-repressor molecules. This leads to direct or indirect interaction between DNMT1 and RUNX1-MTG8 in a complex that includes co-repressors like HDACs, mSIN3a, and N-Cor [103]. Together, these components synergistically silence specific genes, such as IL-3.

(9) HESX1 (HESX homeobox 1)

In early embryonic development, numerous genes are precisely and temporally regulated. HESX1 (HESX homeobox 1), a transcription factor, plays a crucial role in controlling the timing of this regulation. It achieves this by repressing HESX1-target genes through a combination of mechanisms: recruiting co-repressors like TLE1 or N-Cor and facilitating specific DNA methylation through an interaction with DNMT1 in a HESX1/DNMT1 manner [105].

(10) DAXX

DNMT1-containing complexes can target specific TF (transcription factor)-response elements through indirect interactions with TFs. For example, the presence of the transcriptional repressor DAXX (Death-domain associated protein) leads to the silencing of ReIB target genes that this TF typically activates. This repression involves the recruitment of DNMT1, primarily through the indirect interactions of DAXX with DNMT1 and Rel B. Additionally, HDAC2 is recruited by these promoters to complete the repression process. Similarly, in lymphoblastic leukemia, P53 is required to recruit DAXX and DNMT1 to the RASSAF1A promoter, leading to its methylation [106].

(11) CHAF1A

Neuroblastoma originates from the embryonal neural crest due to a differentiation block. Long-term survival correlates inversely with differentiation levels, and treatments like retinoic acid offer modest improvements. This study identifies CHAF1A, a histone chaperone and epigenetic regulator, as crucial in maintaining the aggressive dedifferentiated state of neuroblastoma. CHAF1A influences H3K9 trimethylation of key genes involved in proliferation, survival, and differentiation, and its high expression is linked to poor prognosis. Loss of CHAF1A promotes neuronal differentiation and alters oncogenic signaling pathways and glycolytic metabolism. Thus, CHAF1A significantly restricts neural crest differentiation and the pathogenesis of high-risk neuroblastoma [107].

## 5. DNMT1 Regulators

(1) MEK/ERK

Mitogen-activated protein kinase (MAPK) and extracellular signal-regulated kinase (ERK), pivotal components within the MAPK signaling pathway, are renowned for their multifaceted roles in regulating cell proliferation, differentiation, and survival [108]. They have also been implicated in influencing DNA methylation indirectly through the modulation of DNMT activity or their downstream signaling effects [109]. For example, the activation of the MAPK/ERK pathway in human cancer cells has been demonstrated to enhance the expression of DNMT1, a key enzyme in maintaining DNA methylation patterns during replication [110]. This regulation can contribute to increased DNA methylation and the repression of TSGs. However, there is limited direct evidence supporting that ERK proteins bind DNA directly or facilitate the recruitment of DNMTs to CpG-rich regions [59]. Recent findings suggest that MAPK/ERK signaling can regulate DNMT1 expression through intermediary signaling pathways, as seen in endothelial cells. Specifically, Kim et al. (2023) reported that fibroblast growth factor (FGF)-induced MAPK/ERK signaling regulates DNMT1 expression, impacting cell adhesion molecule (CAM) expression and T-cell chemokine expression [59]. In addition, in systemic lupus erythematosus (SLE), DNA hypomethylation in immune cells has been associated with abnormal MEK/ERK pathway activity [111]. Studies suggest reduced DNA methylation in SLE may involve suppressing DNMT1 expression via dysregulated signaling pathways. For instance, chemical inhibition or siRNA-mediated silencing of PP2Ac in T cells was shown to sustain MEK and ERK phosphorylation upon stimulation with phorbol 12-myristate 13-acetate and ionomycin [112]. This suppression of PP2Ac was linked to enhanced DNMT enzyme activity, increased DNA methylation, and reduced expression of methylation-sensitive genes. These mechanisms were further corroborated in SLE T cells, where PP2Ac suppression amplified MEK/ERK phosphorylation, upregulated DNMT1 expression, and repressed the methylation-sensitive CD70 gene [112]. These findings highlight the regulatory role of MAPK/ERK signaling in DNMT1 expression and the epigenetic landscape contributing to SLE pathogenesis. However, the precise mechanisms by which upstream signaling modulates DNMT1 and DNA methylation remain an active area of investigation.

(2) STAT3

The repression of the PTPN6 gene occurs through the recruitment of a complex involving DNMT1, phosphorylated STAT3, and HDAC1 to the STAT3 boxes in the PTPN6 promoter [103]. Additionally, the acetylation of STAT3 at K685 (K685ac) increases in melanoma, triple-negative breast cancer (BC), and colon cancer compared to normal tissue. This acetylation is associated with a distinct DNA methylation profile mediated by DNMT1 [113]. A mutation in STAT3 at the K685R position disrupts the interaction between DNMT1 and STAT3, thereby restoring the expression of related genes. Furthermore, inhibiting the DNMT1/STAT3 interaction using peptide competitors significantly reduces glioma cell proliferation.

(3) MicroRNAs

miR-148a targets DNMT1 and reduces its levels in cancer, whereas miR-29 Family is known as a repressor of DNMT3A and DNMT3B expression by directly targeting their mRNAs [114]. miR-29b directly targeted DNMT3A and DNMT3B via their 3′ untranslated regions (UTRs) and indirectly downregulated DNMT1 by targeting Sp1, a pivotal transcription factor responsible for DNMT1 expression [115]. Overexpression of miR-29b in MV4-11 and Kasumi-1 significantly reduced the mRNA and protein levels of DNMT1, DNMT3A, and DNMT3B [116]. This reduction led to a global decrease in DNA methylation and, notably, the re-expression of the TSG p15 through promoter DNA hypomethylation. Interestingly, treatment with the HDAC1 inhibitor AR-42 in Kasumi-1 and NB4 cells and leukemic blasts obtained from primary AML patients resulted in the downregulation of miR-29b targets, including Sp1, DNMT1, DNMT3A, and DNMT3B. The combination of AR-42 and decitabine displayed a synergistic anti-leukemic effect in both in vitro and in vivo models [117]. This result holds the promise of using miR-29b oligonucleotides as viable hypomethylating agents that can reduce DNMT expression. Additionally, evaluating the levels of miR-29b in conjunction with DNMT expression might provide a valuable way to predict results in clinical trials involving decitabine for patients. In glioblastoma and breast cancer, DNMT1 controls the methylation of the miRNA-20a promoter, thereby regulating the proliferation, invasion, and apoptosis of tumor cells [118,119]. Higher expression of miR-375 (a significant tumor suppressor) positively correlates with improved overall and disease-free survival. The reduced expression of miR-375 has been attributed to DNA hypermethylation of the promoter region precursor-miR-375 [120]. Overexpression of miR-375 led to the inhibition of proliferation and colony formation and resulted in reduced tumor size and extended survival in a leukemia xenograft mouse model. An intriguing aspect of this regulatory mechanism is also reported in the homeobox protein HOXB3, which induces DNMT3B expression. DNMT3B binds to the promoter region pre-miR-375, resulting in heightened DNA hypermethylation and decreased miR-375 expression within HL-60 and THP1. This research uncovers a new regulatory network involving miR-375, HOXB3, CDCA3, and DNMT3B in AML, providing insights into the complex mechanisms underlying leukemogenesis.

(4) Circular RNAs

Circular RNAs (circRNAs) are intriguing molecules known for their unique structure, featuring closed loops formed through back-splicing during RNA processing. These non-coding RNAs have been found to play a variety of roles in biological processes, including the sequestration of microRNAs, modulation of gene expression, and even serving as templates for protein synthesis [121]. In the context of breast cancer, the circular RNA circ-*Dnmt1* is significantly upregulated in multiple breast cancer cell lines and patients diagnosed with breast carcinoma, and silenced circ-*Dnmt1* decreases cell proliferation and survival rates. Conversely, introducing circ-*Dnmt1* increased these processes by promoting autophagy. Circ-*Dnmt1* was shown to interact with both p53 and AUF1, influencing their movement into the nucleus. This nuclear translocation of p53 initiated autophagy, while AUF1 contributed to stabilizing *Dnmt1* mRNA, which boosted DNMT1 protein levels [45].

## 6. DNMT1 Inhibitor

DNMT inhibitors are activated through the conversion into 5-azacitidine triphosphate by nucleotide reductase and uridine cytidine kinase. This process allows them to integrate into DNA as analogs of cytosine during replication, resulting in the inhibition of cytosine methylation. The documented effects of this inhibition lead to the degradation of DNMTs [122]. The inhibition of DNMTs can significantly influence gene expression and tumorigenesis, showing promise in the reactivation of TSGs, inducing apoptosis, reducing metastasis, enhancing tumor immunogenicity, and augmenting combination therapy with other agents [123]. For instance, decreased DNA methylation levels resulting from DNMT suppression can reactivate TSGs such as CDKN2A at chromosome 9, band p21.3, and CDKN2B [124]. Moreover, DNMT inhibitors lead to the formation of DNA-DNMT protein adducts, which can result in DNA damage and trigger a DNA damage response during replication [125]. The proposed mechanism for the anticancer activity of DNMT inhibitors involves the reactivation of TSGs via CpG demethylation; however, clinical translation has encountered challenges, particularly with solid tumors [126]. Despite these promising mechanisms, a strong correlation between demethylation, tumor suppressor reactivation, and clinical efficacy in human malignancies remains elusive [127].

DNMT inhibitors play a significant role in promoting immune responses by modulating immunosuppressive cells. One of their key actions is inhibiting T lymphocyte differentiation into regulatory T cells, which is achieved by regulating Foxp3 expression [128]. Additionally, DNMT inhibitors counteract the immunosuppressive effects of myeloid-derived suppressor cells by inducing their differentiation into dendritic cells [129]. These inhibitors also enhance the differentiation of CD4^+^ T cells into Th1 CD4^+^ T cells by boosting IL-17 secretion. Moreover, they enable the expression of the killer cell immunoglobulin-like receptor (KIR) on NK cells through DNMTi-induced demethylation, facilitating the recognition of aberrant tumor cells via MHC I binding [130]. Notably, azacitidine (5-azacytidine) has increased the expression of T-cell chemokines, such as Cxcl9 and Cxcl10. These chemokines are critical for enhancing immune responses and have implications for combination therapy with checkpoint inhibitors [2,59]. They contribute to the recruitment of immune cells and the anti-tumor immune response, which can also influence anti-tumor angiogenesis [131]. Preclinical studies suggest that DNMT1 inhibition can upregulate PD-L1 expression, cancer-testis antigens (CTAs), and other TAAs, presenting promising targets for cancer immunotherapy [132].

Currently, the most popular cytidine analogs that are FDA-approved include decitabine (5-aza-2′-deoxycytidine or DAC), azacitidine (5-azacytidine), and guadecitabine. While decitabine and azacitidine are established in clinical use, guadecitabine is undergoing multiple clinical trials [133]. Decitabine, which received FDA approval in 2006, is crucial in treating myelodysplastic syndromes (MDS) and various cancers. Upon administration, decitabine is metabolically converted to a potent cytosine analog called 5-aza-2′-deoxycytidine triphosphate via deoxycytidine kinase. Remarkably, its inhibitory effect on DNMTs is 30 times more potent than that of azacitidine [134]. The drug exhibits diverse effects depending on its concentration. Decitabine can disrupt normal DNA structure at elevated levels, leading to cell death. Conversely, it effectively inactivates DNMTs at lower doses without inducing cell death [135]. Notably, combined with the histone deacetylase inhibitor and decitabine, it enhances apoptosis in thoracic cancer cells by increasing the acetylation of histones H3 and H4 [136]. In in vivo studies, decitabine has demonstrated efficacy in regressing EL4 tumor growth in C57BL/6 mice [137]. In clinical trials, common side effects were reported, and complete tumor responses in patients with AML and MDS present several challenges, largely due to the drug’s biodegradability [138,139].

Guadecitabine exhibits enhanced pharmacokinetics and stability, positioning it as a promising DNMT inhibitor for solid tumors and hematological malignancies [140,141]. However, due to its unique phosphodiester bond, guadecitabine is resistant to degradation by cytidine deaminase, significantly reducing methylation levels in T24 and HCT116 cells while decreasing DNMT1 expression in vitro [142]. Historically, previously developed DNMT inhibitors were rapidly degraded by cytidine deaminase in various organs such as the liver, spleen, and intestinal epithelium, resulting in a short plasma half-life. Guadecitabine successfully addresses these challenges through its innovative chemical structure and pharmacological design [142]. Guadecitabine is undergoing clinical evaluation in multiple cancer trials (NCT03603964, NCT02998567, NCT0330896, and NCT04340843). Initial Phase I and II studies have demonstrated that it is well-tolerated and shows significant response rates in patients with relapsed or refractory AML, as well as in treatment-naïve elderly AML patients [143]. Furthermore, researchers have identified predictors of clinical response and extended survival, including somatic mutations and the blood demethylation rate.

In addition, guadecitabine is a drug designed to enhance interferon-mediated signaling, improve MHC I antigen presentation, and potentially synergize with immune checkpoint inhibition. In human xenotransplantation tumor models, guadecitabine has demonstrated its ability to diminish DNA methylation in the promoter region of the p16 gene [58]. This action enhances interferon signaling and class I antigen presentation, which is crucial for an effective anti-tumor immune response. Furthermore, the combination of guadecitabine with a histone deacetylase (HDAC) inhibitor (MS275) has been shown to promote the reversal of epithelial–mesenchymal transition (EMT) in triple-negative breast cancer (TNBC) cells by facilitating the trimethylation of lysine-27 on histone H3 (H3K27me3) [142]. Notably, higher levels of H3K27me3 correlate positively with patient survival rates in breast cancer, indicating that the combination of guadecitabine and HDAC inhibitors may enhance survival outcomes [144]. Current clinical trials are also addressing the limitations of azacitidine and decitabine in treating AML and Myelodysplastic Syndromes (MDS). These studies will evaluate guadecitabine’s efficacy and explore novel DNMT inhibitors and combination therapies [145]. Significantly, guadecitabine enhances the immunogenicity of tumor cells and upregulates MHC I by inhibiting DNA hypermethylation of the MHC I promoter, thus improving the presentation of TAAs and enhancing immunogenicity [57]. Additionally, guadecitabine promotes the expression of cancer-testis antigens, which help CTLs distinguish tumor cells from healthy cells. The NIBIT-M4 Phase 1b trial highlighted the combination of guadecitabine and ipilimumab, an anti-CTLA-4 antibody, in patients with unresectable melanoma. This study showed promising long-term outcomes, reporting a 5-year overall survival rate of 28.9% and a median response duration of 20.6 months. Mechanistic analyses revealed that guadecitabine reactivated immune pathways by promoting the expression of endogenous retroviral elements (ERVs) and innate immunity-related genes. This reactivation enhanced the effects of adaptive immune responses driven by ipilimumab. Responders showed increased CD8^+^ T-cell infiltration, robust genetic immunoediting, and activation of immune processes, while non-responders exhibited tumor profiles characterized by proliferation and epithelial-to-mesenchymal transition (EMT). Furthermore, developing an integrated immunoediting and adaptive immunity index successfully stratified patients and predicted clinical outcomes, offering valuable insights into the mechanisms of response and resistance in therapeutic settings. These findings underscore the potential of combining epigenetic and immune checkpoint therapies for advanced melanoma, paving the way for further research and clinical applications [146].

Aza-T-dCyd is structurally similar to aza-dCyd, which is modified by adding a nitrogen atom at the 5-position of the pyrimidine ring, replacing a carbon atom. It is distinguished by its oral bioavailability and lower toxicity compared to aza-dCyd, making it a candidate for evaluating DNMT1 depletion in various diseases. Unlike aza-Cyd and aza-dCyd, aza-T-dCyd exhibits a more extensive therapeutic index in mice and can effectively deplete DNMT1 with reduced toxicity, allowing extended treatment in vivo [147,148].

## 7. DNMT Family and Cancer

Altered DNA methylation is a major epigenetic abnormality in tumors [149]. The overexpression of DNMTs, specifically DNMT1, DNMT3A, and DNMT3B, leads to hypermethylation and the activation of oncogenic processes in various tumors [150]. Increased levels of DNMT1 are closely associated with abnormal DNA methylation in solid tumors, which can result in lymph node metastasis and poor patient prognosis [150]. Likewise, elevated expressions of DNMT3A and DNMT3B have been observed in many patient samples, with heightened levels of DNMT3A contributing to the development of hepatocellular carcinoma [42,151]. High expression levels of DNMT3B, in conjunction with the CTCF protein, play a critical role in the epigenetic inactivation of BRCA1 in sporadic breast tumors [42]. DNMT3B is also essential for the growth of colonic microadenomas [116,152]. Furthermore, its overexpression is closely linked to high CpG island methylator phenotype (CIMP-high) levels in colon cancers.

Hence, hypermethylation causes the silencing of cell adhesion genes, such as CDH1, in gastric cancer. Similarly, in primary non-small cell lung cancer, the gene CDH13 is affected, which promotes invasion and metastasis in these tumors [153,154,155,156]. Furthermore, genes associated with DNA repair are often found to be hypermethylated in tumors. In gastric cancer, the hypermethylation of the MLH1 gene leads to defective DNA mismatch repair, facilitating cancer progression [43]. The apoptosis pathway, regulated by death-associated protein kinase 1 (DAPK1) and its mediator TMS1, is also crucial; hypermethylation results in the silencing of TMS1, which promotes the proliferation of breast cancer cells, thereby contributing to disease progression [157]. The dysregulation of DNA methylation in tumor cells is predominantly characterized by widespread hypomethylation and localized hypermethylation. A decrease in global DNA methylation levels is often linked to the activation of proto-oncogenes. At the same time, hypomethylation is frequently observed in solid tumors, with more severe malignant characteristics correlating with higher levels of methylation. Elevated methylation levels are typically associated with silencing TSGs [115,158]. The mechanisms behind DNA hypermethylation in tumors are diverse; multiple genes, such as APC, DAPK1, MGMT, P16, and RASSF1, can be affected simultaneously within a single tumor [81,99,150].

Somatic mutations in DNMTs play a significant role in the malignant transformation of various tumors (See Table 2) [150]. Mutations in DNMT1 are common in colon tumors, while DNMT3A mutations are prevalent in hematological malignancies like AML, MDS, and ETP-ALL [159]. These DNMT3A mutations are associated with disease aggressiveness and resistance to treatment. Mice expressing the DNMT3A Arg882 mutant protein develop chronic myelomonocytic leukemia with thrombocytosis [160,161]. Additionally, mutations in the catalytic domain of DNMT3A significantly reduce its enzymatic activity, leading to hypomethylation of HOX family genes [55,56]. DNMT3B mutations are rarely found in AML; increased levels of DNMT3B expression are linked to poor outcomes, especially in pediatric AML patients [116]. Interestingly, DNMT3B may act as a tumor suppressor in certain subtypes of AML, such as MLL-AF9 AML and inv (16) (p13;q22) AML, highlighting a complex role in leukemia development [116]. Moreover, DNMT3B overexpression and DNA hypermethylation have been observed in T-cell acute lymphoblastic leukemia and Burkitt’s lymphoma, which underscores its oncogenic role in hematological malignancies through the hypermethylation of TSGs [159].

Hypomethylation by suppressing DNMT1 activity occurs throughout the genome, reducing 5mC, particularly in gene coding regions and satellite repeats.

These alterations can result in mitotic recombination, copy number deletions, chromosomal rearrangements, and even the loss of genomic imprinting. Reducing or deleting DNMT1 can cause significant genome-wide hypomethylation and chromosomal instability [125]. In development, a study using an in vivo mouse model with embryonically inactive DNMT3A and DNMT3B has shown that the deletion of de novo methyltransferases leads to lethal phenotypes [11]. Recently, research utilizing conditional knockout technology has illuminated the role of de novo methyltransferases in hematopoiesis. Deleting Dnmt3a in adult mice results in the proliferation of hematopoietic progenitors. This abnormality is associated with the accelerated development of malignancies driven by mutated NRAS or FLT3-ITD [173]. Furthermore, introducing c-Kit variants into a *Dnmt3a*-deficient background can induce acute leukemia. In tumors, inactivation of DNMT3A has also been linked to the development of peripheral T cell lymphoma (PTCL) and lung tumors, suggesting that DNMT3A may act as TSGs. Additionally, studies indicate that DNMT3B is a tumor suppressor in Myc-induced lymphomas and MLL-AF9-driven AML [152,158]. The loss of maintenance methyltransferase activity is also connected to carcinogenesis. For instance, the deletion of DNMT1 leads to DNA demethylation. It is crucial in preventing and maintaining T-cell lymphoma, contributing to abnormal methylation through de novo and maintenance mechanisms. Consequently, the deletion of genes encoding DNMTs is implicated in tumor development.

The regulation of inflammation by DNMT1 occurs across various cell types, including immune, endothelial, and epithelial cells [4]. DNMT1 is essential for maintaining specific methylation sites within cytokine promoters, effectively suppressing their expression. In immune cells, DNMT1 plays a critical role in controlling T-cell activation and differentiation, with its expression significantly upregulated in activated T cells [174]. Inhibiting DNMT1 activity has been associated with decreased T-cell proliferation and cytokine production [59,174]. Similarly, demethylation of specific gene loci in T and B lymphocytes is linked to several cellular processes. These include the regulation of immunoglobulin and T-cell receptor (TCR) gene accessibility, lineage-specific expression of proteins such as CD4, CD8, FcγR, and CD21, and the transcriptional regulation of key cytokine genes like interferon-γ (IFN-γ), IL-3, IL-4, and IL-5 [174]. Furthermore, in macrophages, the enzymes DNMT3A and DNMT3B are crucial for establishing new DNA methylation patterns, while DNMT1 is essential for maintaining these modifications during cell division. Furthermore, the methylation landscapes shaped by these DNMT enzymes directly influence macrophage polarization, thereby affecting the inflammatory response [175]. The altered methylation status of PPAR1 promoter by DNMT3B inhibits the expression of M2 (anti-inflammatory) macrophage genes and drives macrophages toward a more inflammatory state [176]. Pharmacological inhibition of DNMT activity, with agents like 5-aza-2′-deoxycytidine, can reverse these processes by promoting M2 activation and reducing inflammation driven by macrophages [176,177]. Integrating DNA methylation and gene expression analyses may help researchers uncover key factors in reprogramming tumor-associated macrophages (TAM), potentially leading to novel therapeutic targets and developing new predictive and diagnostic biomarkers. Alterations in DNA methylation levels can significantly impact the production of critical cytokines, including IL-10 and TGF-β, which are crucial in tumor development [178,179,180]. Specific CpG dinucleotides within the promoter regions of these genes have been identified as targets for DNMT1-mediated DNA methylation. The methylation of these sites correlates with reduced expression of CXCL9 and CXCL10, which is associated with enhanced immune cell infiltration into the tumor microenvironment and a subsequent reduction in tumor growth [2,59].

**Figure 3:**
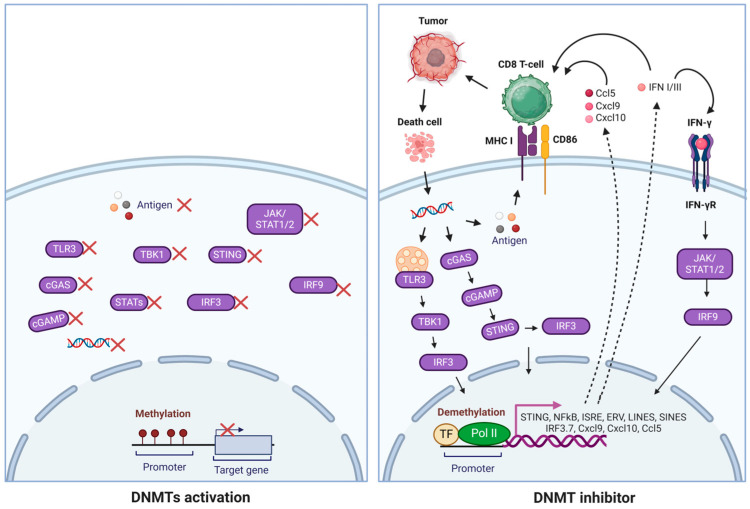
Mechanisms of DNMT inhibitors in reactivating immune responses. DNMT inhibitors promoted the reactivation of genes involved in cell cycle regulation, apoptosis, DNA repair, T-cell chemokines, ERVs, and IFN-γ signatures, activating innate and adaptive immune responses. Tumor DNA is transported to the cytoplasm, where it triggers the cGAS/STING pathway, leading to the expression of interferons, MHC I, and co-stimulatory molecules such as CD86. Additionally, STING activates TBK1, which then recruits IRF3 for phosphorylation. Once phosphorylated, IRF3 dimerizes and translocates to the nucleus, which works with NF-kB to initiate the expression of type I interferons and other immunomodulatory molecules.

DNA methylation can enhance immunotherapies by activating immune-stimulatory signaling and promoters encoded by ERVs [181]. Recent deep RNA sequencing data indicated that thousands of DNMT1 and HDAC inhibitors induce novel polyadenylated transcripts (TINPATs) [181]. Further analysis identified the human leukocyte antigen (HLA) presentation of 45 validated treatment-induced neopeptides (t-neuropeptides) from TINPATs, suggesting their potential to provoke T-cell responses against cancer cells. The newly detected t-neopeptides originated from decitabine-treated AML patients, strongly indicating the potential role of ERV-derived neo-antigens in epigenetics and immunotherapies [182,183]. Another experiment explored the potential role of DNMT1 in supporting tumor antigenicity. A significant challenge in cancer biology is overcoming immune escape and resistance to T-cell-based immunotherapy. A comprehensive genome-wide DNA methylation study aimed at identifying DNA methylation-dependent gene silencing revealed hypermethylation of the cGAS and STING genes, severely impairing STING function in human melanoma. This epigenetically altered suppression can be reversed by pharmacological inhibition of DNA methylation, restoring STING functionality (Figure 3) [183]. When the drug effect of a DNMT1 inhibitor induces the activation of CD8^+^ T cells, which kill cancer cells, the dying tumor cells release double-stranded DNA (dsDNA) into the tumor microenvironment, where cGAS (cyclic GMP-AMP synthase) detects and binds dsDNA, catalyzing the production of cyclic 2′3′-GMP-AMP (cGAMP) from ATP and GTP. cGAMP binds to the STING (Stimulator of Interferon Genes) protein on the endoplasmic reticulum, leading to STING’s activation and movement to the Golgi apparatus. Activated STING recruits TBK1, which phosphorylates IRF3, prompting its dimerization and translocation to the nucleus. IRF3 and other factors like NF-κB induce type I interferons and inflammatory cytokines crucial for antiviral responses and immune modulation. Furthermore, the restored STING functionality enhances the antigenicity of melanoma cells by increasing MHC I expression, thereby improving their recognition and elimination by cytotoxic T cells (Figure 3) [183].

## 8. Conclusions and Future Perspectives

Aberrant DNA methylation is the most common epigenetic mark and a promising target for therapeutic interventions in various human cancers. It is well-accepted that increasing genome-wide DNA methylation correlates with aggressive brain tumors and is tightly regulated by DNMTs [184]. Recent experiments and clinical trials of combination therapies, such as adjuvant therapy, chemotherapies, and immunotherapies, have shown significant progress [141,185]. For example, the recent combination therapy for MAPK or BRAF inhibitor and DNMT1 inhibitor demonstrates strong synergistic effects [186,187]. Future studies are vital for understanding the molecular mechanisms of MAPK/ERK signal transduction pathway and drug action mechanisms and carefully investigating side effects in clinical settings with larger cohorts [36,186,187]. RNAseq and multi-omics data will provide valuable information for developing innovative approaches for brain tumors, especially glioma (GBM). In addition, CRISPR-based gene editing tools will open new avenues for precise modification of DNA methylation at specific sites. Using DNA methylation signatures to build a predictive model for brain metastasis development integrates clinical factors to provide patient-specific brain metastasis risk probability. It will enhance personalized treatment for better patient outcomes. Notably, the strategy for genetic deletion of Dnmt1 in endothelial cells utilizing a Ve-Cadherin inducible system has made significant progress in understanding the role of DNMT1 in tumor angiogenesis and immune regulation in the tumor microenvironment [59]. Recent clinical data from the five-year follow-up and integrated multi-omic analysis of the NIBIT-M4 Phase 1b trial demonstrate that guadecitabine promotes and leads to the re-expression of immunomodulatory retroviruses and repetitive elements, enhancing innate immune pathways, including interferons, NF-kB, and TLR signaling. Guadecitabine also modulates the tumor microenvironment, highlighting the synergy between epigenetic therapy and immunotherapy. This represents a promising new approach for treating advanced melanoma. In addition, a second generation of nucleoside analogs, 5-aza-T-dCyd, has been shown to enhance pharmacological properties against solid tumors [135,147,148,177]. Furthermore, recently developed 5-aza-4′-thiol-2′-b-fluoro-2′-deoxycytidine can significantly influence the molecule’s stability, bioavailability, and/or epigenetic activity [188]. The genome-wide analysis of the accuracy of DNA methylation, potential side effects, and long-term outcomes of using 5-aza-T-dCyd and 5-aza-4′-thiol-2′-b-fluoro-2′-deoxycytidine in clinical settings requires thorough investigation to ensure patient safety and treatment efficacy.

Enhancing the specificity of DNMT (DNA methyltransferase) inhibition at specific promoters is a challenging yet crucial objective in epigenetic therapy and research. Targeting DNMT inhibitors to precise loci using DNA-binding modules can address the lack of promoter specificity in existing DNMT inhibitors. CRISPR/dCas9-mediated targeting is a promising approach. Catalytically inactive Cas9, called dCas9, can be paired with specific domains to promote DNA demethylation. This is achieved by fusing dCas9 with elements such as the TET1 catalytic domain or inhibitors of DNMT. Using a guide RNA (gRNA) to pinpoint a particular promoter region, dCas9 is directed to bring the demethylating agent to that site. Protein docking strategies engineer dCas9 to recruit inhibitory peptides or small molecules that block DNMT at the targeted promoter. These approaches boast impressive sequence specificity and significantly reduce the risk of off-target effects, as they operate solely near where they bind [58]. The other approach uses zinc-finger or TALE proteins. Zinc-finger proteins fused to demethylating domains or engineered zinc-finger (ZF) arrays can target specific 9–18 bp sequences for high specificity at promoters. A ZF domain linked to a TET demethylase or DNMT inhibitory domain can enable localized demethylation [189]. Transcription activator-like effectors (TALEs) can be programmed to recognize specific DNA sequences. By attaching demethylating modules or DNMT inhibitors, TALEs also allow targeted epigenetic editing at promoters [190,191]. Recent advancements in epigenetic therapy have focused on using CRISPR/dCas9 and engineered protein tools like zinc-finger and TALE proteins to precisely inhibit DNMT at specific promoters, enhancing specificity and minimizing off-target effects.

Beyond tumors, DNMT inhibitors could play a role in restoring normal gene expression in neurodegenerative and psychiatric disorders. That could benefit regenerative therapies using embryonic stem cells and induced pluripotent stem cells (iPSCs). Future directions should focus on developing DNMT inhibitors that are less toxic and specifically target different DNMT isoforms and cancer cells. Combining promising novel DNMT1 inhibitors with other therapeutic strategies, such as chemotherapy, radiotherapy, vaccines, immune checkpoint inhibitors, and CAR-T therapies, may yield effective synergistic responses in cancer treatment.

## Figures and Tables

**Table 1:** DNMT knockout phenotypes.

	Mouse/Cell	Survival	Development	Reference	*E* *tc*
*Dnmt1*	Mouse	Embryonically lethal	Defect	[17]	Global loss of DNA methylation
*Dnmt2*	Mouse/Fly	Viable and fertile	No defect	[12,13]	Aberrant hematopoiesis
*Dnmt3*	Mouse	Embryonically lethal	Impaired postnatal development	[10]	
*Dnmt3b*	Mouse	Embryonically lethal	Defect	[11]	
*Dnmt1*	Mouse ES cell			[16]	Died upon the induction of differentiation
*Dnmt1*	Human ES cell			[18,19]	Died upon the induction of differentiation
Triple knockout (*Dnmt1, Dnmt3a, Dnmt3b*)	Cell			[15]	Proliferation

**Table 2:** Malignant transformation in DNMT family.

	Mutation	Location	Effects	Cancer Association	Ref
DNMT1	Somatic Mutations	Mutations in coding exons of the DNMT1 gene	Genomic instability, loss of DNA methylation control	Colorectal cancers Lung cancer	[154,162]
DNMT1	Catalytic Domain Mutations	Catalytic domain	Loss of function, global hypomethylation, oncogenic activation	Hematologic malignancies, Solid tumors	[163,164]
DNMT1	Splice-Site Mutations	Splice sites	Abnormal splicing, leading to truncated proteins with altered function	AML, MDS, Leukemia	[165]
DNMT1	Deletion/Overexpression	Coding exons	Significantly suppressed tumor formation/Tumorigenesis	Mammary tumor, Breast cancer, Pancreas/Liver	[162,166,167]
DNMT2	Deletion	tRNA methyltransferase domain	Promotes the proliferation, colony formation, and metastasis of hepatocellular carcinoma cells	Hepatocellular carcinoma	[67]
DNMT3A	R882H, R882C, R882P, R882S	Catalytic domain	Dominant-negative inhibition, genome-wide hypomethylation, AML association	Acute Myeloid Leukemia (AML)/MDS	[168,169,170]
DNMT3A	Catalytic domain mutations	C-terminal domain	Loss of function, reduced enzymatic activity, hypomethylation of TSGs	AML, Myelodysplastic Syndromes (MDS)	[160,171]
DNMT3B	Frameshift mutations	Various coding regions	Protein truncation, loss of enzymatic function, genomic instability	Lymphoma	[72,172]
